# Developing Affordable, Portable and Simplistic Diagnostic Sensors to Improve Access to Care

**DOI:** 10.3390/s22031181

**Published:** 2022-02-04

**Authors:** Nikki L. Hafezi, Farhad Hafezi

**Affiliations:** 1 Light for Sight Foundation, 8953 Zurich, Switzerland; farhad@hafezi.ch; 2 GroupAdvance Consulting, 6300 Zug, Switzerland; 3 ELZA Institute AG, 8953 Zurich, Switzerland; 4 EMAGine AG, 6300 Zug, Switzerland

**Keywords:** keratoconus, cornea, topography, diagnostic, access, ophthalmology, crosslinking, screening, prevention, LMIC

## Abstract

Ophthalmology is a highly technical specialty, especially in the area of diagnostic equipment. While the field is innovative, the access to cutting-edge technology is limited with reference to the global population. A significant way to improve overall healthcare is to understand the needs and possibilities of all possible consumers when developing sophisticated and accurate medical devices. The Smartphone-based Keratograph (SBK), is an example of a new project that uses real world feedback, addresses an unmet medical need, and implements commercially available components to create a device that is affordable, portable and simplistic to operate. The long-term goal of the SBK is to collect data from users for supervised machine-learning. This machine-learning aspect will ultimately aid in the development of an artificial intelligence device to enable even earlier detection of keratoconus, especially in children and adolescents. Again, the ultimate goal of any medical device should be to improve patient care, and to make a significant improvement on vision healthcare for the global population, providing access to this technology is essential.

Financial disclosure: Nikki Hafezi is CEO of EMAGine AG, a company producing a CXL device. Farhad Hafezi holds a patent on a UV light source (PCT/CH 2012/000090).

Modern healthcare is technology-driven, and consequently, access is a major challenge for addressing unmet medical needs. There is a rapidly growing disparity in clinical medicine between high income, low middle income and low income (HIC, LMIC, LIC) countries [1]. The dichotomy is that, on the one hand, cutting-edge devices are constantly evolving to allow for highly precise and early diagnosis as well as surgical treatments of rare diseases, but on the other hand, access to common modern diagnostic devices is scarce and limited for the majority of the global population.

Ophthalmology is a highly technical field of medicine and may serve as an example of this ever-growing diagnostic divide. In most modern ophthalmological clinics, diagnostic and therapeutic devices are highly sophisticated but stationary and costly. These devices ensure the highest level of care of both common and rare ophthalmic conditions. However, this level of healthcare technology is unavailable for the vast majority of global ophthalmologists and their patients. Rather, the common slit lamp, developed by Gullstrand in 1911, remains the only piece of equipment to provide ophthalmic healthcare [2]. This basic diagnostic tool emits a sheet of light into the eye and functions as a biomicroscope. While other supplementary devices help improve the features of the slit lamp, ultimately, the diagnosis of diseases is subjective to the experience and knowledge of the treating clinician.

An unmet need in our field is the diagnosis and treatment of keratoconus, a corneal disease which is the leading cause for preventable severe visual impairment in children and adolescents globally [3]. Unlike cataract and glaucoma, knowledge about keratoconus is relatively low among ophthalmic healthcare professionals.

In high-income Countries (HIC), modern diagnostics include device-driven imaging of the cornea using placido-based topographers or Scheimpflug-based corneal tomographers [4]. These tools allow the clinician to detect the disease in its earliest stages in pediatric patients before severe vision loss occurs. Early diagnosis of keratoconus translates into early intervention/treatment. The treatment of keratoconus is performed using a surgical method known as corneal cross-linking (CXL), which is currently conducted in operating theaters [5].

In LMIC, however, modern diagnostic imaging devices are unaffordable and access to operating room infrastructure is limited, especially in remote areas. As a result, enabling technologies that are relatively simplistic to operate, while being portable and affordable, would address the main obstacles in place for better diagnoses and treatments of keratoconus. When defining the unmet needs, portable devices for diagnosis and treatment outside of the large eye clinics are essential, for example in remote areas or schools for screening purposes.

Therefore, the call to action would be to transform the current state of technology by developing devices that would be simple, portable and affordable to enable access to early-stage screening and treatment to help support early intervention for patients with keratoconus. Recently, we developed such a device for the treatment of keratoconus: the C-eye device is a small, portable, yet highly sophisticated UV lamp used to perform corneal cross-linking [6]. The C-eye device can be mounted on a slit lamp and allows for office-based treatment of keratoconus outside an operating theater.

Using the same model of development, we are working on the Smartphone-Based Keratograph Project (SBK), a device that would enable diagnosis of keratoconus. The SBK is funded by two private Swiss foundations and is based on the principle of enabling access to treatment. The concept of the SBK project is to generate measurements that will allow the reader to assess whether the patient’s cornea shows pathological features or not. From this initial screening, the patient can be directed to a specialist for further evaluation. The SBK prototype uses placido-based imaging and a commercially available smartphone to process and store the measurements. The process and means to collect data for later analysis will be explored at a later stage of the project.

The long-term goal of this concept is to collect datasets from the first prototype for supervised machine learning. Next, a second-generation device could differentiate if a patient suffers from keratoconus or other irregularities of the cornea. The goal is to collect enough measurements to eventually create an artificial intelligence-powered medical device to diagnose keratoconus with a single assessment. This medical device would use an interface designed for an unskilled worker to administer outside of a clinical setting. 

As the trend continues in the industry, technology develops at a rapid pace, and therefore, what was considered modern last year becomes obsolete because the focus remains on obtaining an even better solution but with an expensive price tag. Therefore, the real challenge is to change the paradigm. Specifically, the focus should be to convince the industry that instead of investing in an “even better” solution, we should adapt the current technology to develop a more affordable, portable and simple device. Specifically, the business plan for future sensory devices should be based on sales volume of devices and not necessarily on adapting and integrating the latest cutting-edge technology for only an elite number of users.

Another example for such development is the assessment of corneal biomechanics, which saw a number of complex (and costly) technologies emerge such as Brillouin microscopy [7]. Currently, OCT-based approaches are developed which would distinctly reduce the costs related to the technology [8].

In summary, when a company decides to invest into developing sophisticated technology for highly precise and costly surgical treatments, commercialization is the essential goal. Protecting this technology with a strong patent portfolio ensures that the company has sufficient time to recuperate investments and generate a rewarding profit during a limited monopoly period. While the financial motives and strategy are clear, who has access to this technology in the end? Moreover, why can the focus not be on adapting existing technology that is simple, affordable and portable for the masses to use? The answer to this question could stimulate a new type of business model that would be based on sales volume while democratizing access to technology for improved global healthcare. We propose that the improvement of the overall global vision is worth the challenge.

## Data Availability

Not applicable.
